# The expression of DBC1/CCAR2 is associated with poor prognosis of ovarian carcinoma

**DOI:** 10.1186/s13048-015-0129-3

**Published:** 2015-02-15

**Authors:** Dong Hyu Cho, Ho Sung Park, See-Hyoung Park, Kyoung Min Kim, Myoung Ja Chung, Woo Sung Moon, Myoung Jae Kang, Kyu Yun Jang

**Affiliations:** Department of Obstetrics and Gynecology, Chonbuk National University Medical School and Research Institute of Clinical Medicine, Jeonju, Republic of Korea; Departments of Pathology, Chonbuk National University Medical School, Research Institute of Clinical Medicine and Research Institute for Endocrine Sciences, Jeonju, Republic of Korea; Division of Gynecologic Oncology, Department of Obstetrics and Gynecology, Stanford University School of Medicine, Stanford, California USA

**Keywords:** Ovarian neoplasms, Serous carcinoma, Deleted in breast cancer 1, BRCA1, Prognosis

## Abstract

**Background:**

Recent reports have shown that deleted in breast cancer 1 (DBC1/CCAR2) is an indicator of poor prognosis of various human cancers. However, its expression in ovarian carcinoma has not been reported.

**Methods:**

We investigated the immunohistochemical expression of DBC1 and BRCA1 and their prognostic significance in 104 ovarian carcinomas. Survival analyses were performed according to the Kaplan-Meier method, as well as univariate and multivariate Cox proportional hazard regression analysis.

**Results:**

Positive expression of DBC1 and BRCA1 were seen in 63% (66/104) and 44% (46/104) of overall ovarian carcinomas, respectively. DBC1 expression was significantly associated with advanced clinicopathological factors such as high tumor stage, latent distant metastasis, platinum-resistance, elevated serum levels of CA125, high histologic grade, and BRCA1 expression. In the histological subtypes of ovarian carcinomas, DBC1 expression was more common in serous carcinoma (72%, 54/75) than mucinous carcinoma (15%, 3/20). BRCA1 expression was significantly associated with latent distant metastasis, platinum-resistance, and higher histologic grade. In addition, DBC1 expression was significantly associated with shorter overall survival (OS) and relapse-free survival (RFS) in 104 ovarian carcinomas (OS; *P* < 0.001, RFS; *P* < 0.001) and 63 high-grade serous carcinomas (OS; *P* = 0.008, RFS; *P* = 0.023) by univariate analysis. BRCA1 expression was significantly associated with OS and RFS in 104 ovarian carcinomas (OS; *P* = 0.005, RFS; *P* = 0.002) and 75 serous carcinomas (OS; *P* = 0.047, RFS; *P* = 0.038) by univariate analysis. Moreover, DBC1 expression was an independent prognostic indicator for OS in both 104 ovarian carcinomas (*P =* 0.021) and 63 high-grade serous carcinomas (*P =* 0.011) by multivariate analysis.

**Conclusions:**

These results indicate that the expression of DBC1 and BRCA1 are closely related with in the progression of ovarian carcinomas and may have clinical utility in the prediction of prognosis of ovarian carcinomas. Especially, DBC1 expression could be employed as a significant prognostic indicator for ovarian carcinomas especially in high-grade serous carcinomas.

## Background

Deleted in breast cancer 1/cell cycle and apoptosis regulator 2 (DBC1/CCAR2) was named by its deletion at a region 8p22 in breast cancer. Thereafter, DBC1 was re-designated CCAR2 to eliminate possible confusion with *deleted in bladder cancer 1* and because it has partial sequence homology to CCAR1. The deletion of DBC1 in breast cancer suggested it may have a role as a tumor suppressor [1]. Based on data of cBio Cancer Genomics Portal (http://www.cbioportal.org), the deletion of DBC1 has been reported in 2.5% (8/316) to 7.7% (24/311) of ovarian serous carcinomas [2,3]. Especially, the inhibitory role of DBC1 on SIRT1 supported the possibility that DBC1 could be tumor suppressor because SIRT1 inactivates various tumor suppressors, especially p53 [4,5]. However, these findings were followed by conflicting reports, which cast doubt on whether DBC1 is tumor suppressor. In human cancers, the deletion of DBC1 was not a common phenomenon and the balance between SIRT1 and DBC1 was disrupted in human cancers [6]. When the interaction between SIRT1 and DBC1 is week, the depletion of DBC1 makes cells susceptible to UV-induced genotoxic stress [7]. Also, DBC1 inhibits senescence of premalignant cells by disrupting the SUV39H1-SIRT1 complex [8]. Moreover, the expression of both DBC1 and SIRT1 were correlated with advanced clinicopathological characteristics and poor prognosis of human malignant tumors [9-16]. In addition, it has been shown that DBC1 has multiple functions involved in the regulation of cell survival, energy metabolism, and intracellular signal transduction [8,12,17-21]. Therefore, DBC1 might have its own role in tumorigenesis in addition to an inhibitory role for SIRT1.

The tumorigenic role of DBC1, although controversial, is supported by its role in the inhibition of tumor suppressors [8,22] and activation of nuclear receptors with tumorigenic potential [21]. DBC1 inhibits the tumor suppressor BRCA1 by binding to the BRCT domain in breast cancer [22]. Defect of *BRCA1/2* is involved in the development of ovarian carcinomas and defects in *BRCA1/2* are associated with the resistance to platinum-based chemotherapy in ovarian carcinomas [23,24]. Therefore, there is a possibility that DBC1 may be involved in *BRCA1/2*-related progression of ovarian carcinomas. In addition, inhibition of DBC1 reduced the proliferation and invasive potential of gastric cancer cells and squamous cell carcinoma cells [12,20]. The decrease of tumor invasiveness with inhibition of DBC1 was associated with a decrease in epithelial-mesenchymal transition (EMT) signaling [12].

The role of DBC1 as a co-activator of hormone receptors raised the possibility that DBC1 could promote the tumorigenesis of hormone-dependent organs [18,19,21]. In breast carcinoma, DBC1 is expected to be an indicator of poor prognosis and might be involved in resistance to the estrogen receptor-targeted therapies [14]. However, there has been no study to date on the role of DBC1 in ovarian tumorigenesis, its relation to BRCA1/2, and the prognostic significance of DBC1 in ovarian cancers. Therefore, in this study, we evaluated the immunohistochemical expression of DBC1 and BRCA1 and their prognostic significance in 104 ovarian carcinomas.

## Methods

### Patients and tissue samples

One hundred and four ovarian carcinomas diagnosed between November 1996 and August 2008, and the original histologic slides, tissue blocks, and clinical information were available were included in the present study. The age of the patients ranged from 21 to 82 years (median; 54 years). All patients received staging operations and 83 patients received platinum- and taxoid-based adjuvant chemotherapy. Among the 83 patients, platinum-resistance was evaluable in 82 patients and 62 patients were sensitive to platinum-based chemotherapy and 20 patients showed platinum-resistance. Platinum-resistance was evaluated according to the standard Gynecologic Oncology Group criteria [25]. The patients who experienced recurrence or progression of ovarian cancer within the six months of platinum-based chemotherapy were included in platinum-resistant group. Among the 104 ovarian carcinoma patients, 39 patients experienced relapse and 50 patients died from ovarian carcinoma at the endpoint of follow-up. The five- and ten-year overall survival (OS) rates were 57% and 50%, respectively. This study was approved by the institutional review board of Chonbuk National University Hospital. Informed consent was provided according to the Declaration of Helsinki.

All histologic slides and clinicopathologic factors were reviewed according to the criteria of the World Health Organization classification of tumors of female reproductive organs [26]. The ovarian carcinomas included in this study, according to the histologic types, were 75 serous carcinomas (12 low-grade serous carcinomas and 63 high-grade serous carcinomas), 20 mucinous carcinomas, 5 endometrioid carcinomas, 3 clear cell carcinomas, and one malignant Brenner tumor. Tumor stage was reviewed according to the guidelines of the tumor, node, and metastasis staging system of the American Joint Committee on Cancer [27]. Thereafter, the ovarian carcinomas were grouped according to their age (<60 years *versus* ≥ 60 years), tumor stage (I and II *versus* III and IV), tumor size (≤10 cm *versus* > 10 cm), lymph node metastasis (absence *versus* presence), presence of ascites (absence *versus* presence), bilaterality (unilateral *versus* bilateral), presence of latent distant metastasis during follow-up (absence *versus* presence), pre-operative serum level of CA19-9 (normal *versus* elevated, reference value; 0 - 37 U/ml), pre-operative serum level of CA125 (normal *versus* elevated, reference value; 0 - 35 U/ml), and histologic grade (low; grade 1 *versus* high; grade 2 and 3). The duration of follow-up ranged from one to 193 months (median; 70 months).

### Establishment of tissue microarray and immunohistochemical staining

Tissue microarray (TMA) established from the most representative solid area with highest histologic grade after review of original H&E slides. One 5 mm tissue core per case was used for the construction of a TMA. Immunohistochemical staining for DBC1 (1:100, Bethyl Laboratories, Mongomery, TX) and BRCA1 (1:100, Abcam, Cambridge, MA) was performed on 4 μm thick sections on TMA slides. The tissue sections underwent a microwave antigen retrieval procedure in pH 6.0 sodium citrate buffer for 20 minutes. Immunohistochemical analysis was performed by two pathologists (KYJ and KMK) by consensus, without knowledge of the clinicopathological information. For the evaluation of the immunohistochemical staining of DBC1 and BRCA1, the Allred nuclear scoring system was used [28]. The staining intensity was scored as 0 (no staining), 1 (weak staining), 2 (intermediate staining), or 3 (strong staining). The area of staining was scored as 0 (no staining), 1 (1% of the cells stained positive), 2 (2-10% of the cells stained positive), 3 (11-33% of cells stained positive), 4 (34-66% of cells stained positive), or 5 (67-100% of cells stained positive). Thereafter, the sum score was obtained by adding the intensity score and staining area score [13,29], to give maximum sum score of eight and a minimum sum score of zero.

### Statistical analysis

The cut-off point of the immunohistochemical staining score for DBC1 and BRCA1 expression was determined by receiver operating characteristic curve analysis at the highest positive likelihood ratio point for the event of OS. The cut-off points for the sum score for DBC1 and BRCA1 were seven and six, respectively. The immunostaining for DBC1 was considered positive when the sum score was greater than or equal to seven and BRCA1 expression was considered positive when the sum score was greater than or equal to six. The endpoint of interest was OS and relapse-free survival (RFS). The endpoint of follow-up was the date of last contact or date of death of patients through August 2013. OS was calculated as the time from the date of diagnosis to the date of last contact or death from ovarian carcinomas. The patients who were alive at last contact were treated as censored for OS analysis. RFS calculated from the date of diagnosis to the date of last contact, local relapse, latent metastasis, or death from ovarian carcinomas. The patients who were alive at last contact without experience of local relapse or latent metastasis were treated as censored for RFS analysis. Survival analyses were performed according to the Kaplan-Meier method, and univariate and multivariate Cox proportional hazard regression analysis. The association between immunohistochemical positivity of DBC1 expression and other clinicopathological factors potentially predictive of prognosis were analyzed using Pearson’s chi-square test. SPSS software (version 20.0) was used throughout and *P*-values less than 0.05 were considered statistically significant.

## Results

### Association of DBC1 and BRCA1 expression with clinicopathologic characteristics of ovarian carcinoma patients

**Figure 1:**
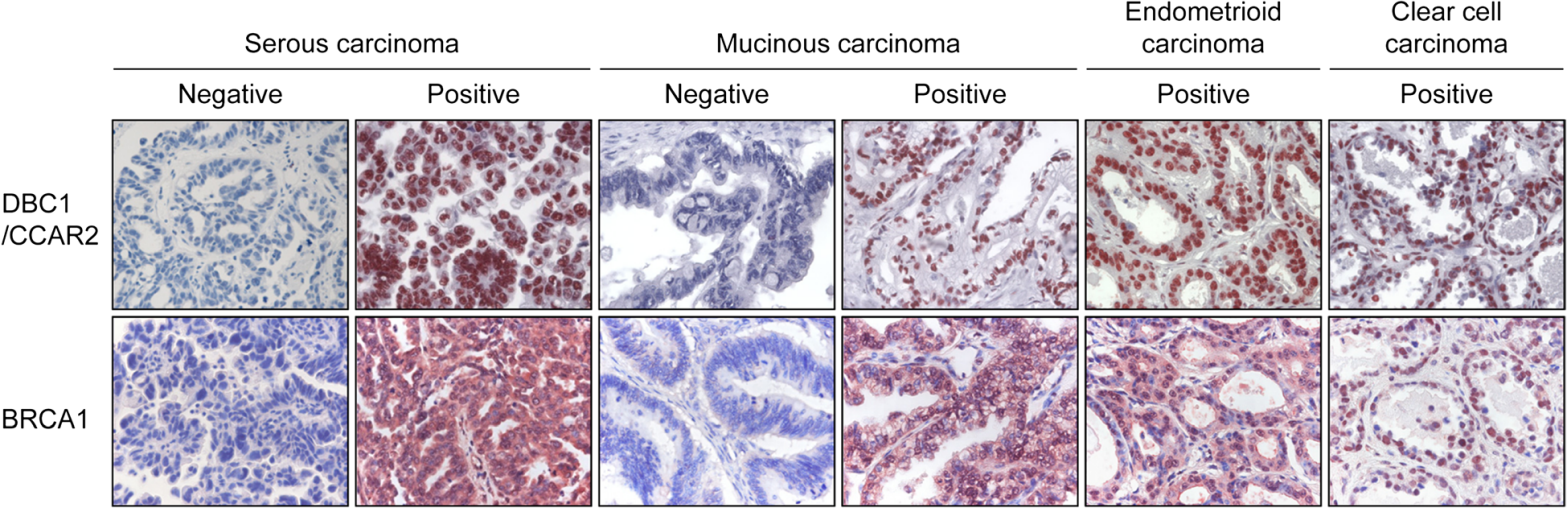
**Immunohistochemical expression of DBC1/CCAR2 and BRCA1 in ovarian carcinomas.** DBC1/CCAR2 is primarily expressed in the nuclei of the tumor cells. BRCA1 is expressed both in the cytoplasm and nuclei of the tumor cells. Original magnification; ×400.

As shown in Figure 1, DBC1 was expressed exclusively in the nuclei of tumor cells and nuclear expression was evaluated for the evaluation of DBC1. BRCA1 was expressed in both the cytoplasm and nuclei; however, we have evaluated only for the nuclear expression [30,31]. The associations between the expression of DBC1, BRCA1, and variable clinicopathologic features are summarized in Table 1. Positive expression of DBC1 and BRCA1 were seen in 63% (66/104) and 44% (46/104) of overall ovarian carcinomas, respectively. Expression of DBC1 was significantly associated with higher tumor stage (*P =* 0.015), bilateral tumors (*P =* 0.008), latent distant metastasis (*P =* 0.016), platinum-resistance (*P =* 0.016), higher pre-operative serum level of CA125 (*P =* 0.004), higher histologic grade (*P <* 0.001), histologic type of ovarian carcinoma (*P <* 0.001), and BRCA1 expression (*P <* 0.001). DBC1 was expressed in 72% (54/75) of serous carcinoma and 100% of endometrioid carcinoma (5/5), clear cell carcinoma (3/3), and the malignant Brenner tumor (1/1). However, only 15% (3/20) of mucinous carcinoma were positive for DBC1. BRCA1 expression was significantly associated with latent distant metastasis (*P =* 0.012), platinum-resistance (*P =* 0.014), and higher histologic grade (*P* = 0.007).

**Table 1:** **Clinicopathological variables and the expression of DBC1 and BRCA1 in ovarian carcinomas**

**Characteristics**		**No.**	**Overall ovarian car**		**cinomas**		**No.**	**Serous c**		**Arcinomas**	
			**DBC1+**	***P***	**BRCA1+**	***P***		**DBC1+**	***P***	**BRCA1+**	***P***
Age, y	<60	71	41 (58%)	0.076	28 (39%)	0.149	45	29 (64%)	0.074	20 (44%)	0.450
	≥60	33	25 (76%)		18 (55%)		30	25 (83%)		16 (53%)	
Stage	I & II	52	27 (52%)	0.015	21 (40%)	0.430	29	18 (62%)	0.128	13 (45%)	0.662
	III & IV	52	39 (75%)		25 (48%)		46	36 (78%)		23 (50%)	
Tumor size, cm	≤10	68	48 (71%)	0.038	31 (46%)	0.702	56	40 (71%)	0.850	26 (46%)	0.64
	>10	36	18 (50%)		15 (42%)		19	14 (74%)		10 (53%)	
LN metastasis	Absence	84	50 (60%)	0.087	37 (44%)	0.939	56	39 (70%)	0.435	28 (50%)	0.552
	Presence	20	16 (80%)		9 (45%)		19	15 (79%)		8 (42%)	
Ascites	Absence	71	41 (58%)	0.076	28 (39%)	0.149	45	31 (69%)	0.462	18 (40%)	0.089
	Presence	33	25 (76%)		18 (55%)		30	23 (77%)		18 (60%)	
Bilaterality	Unilateral	59	31 (53%)	0.008	24 (41%)	0.404	34	21 (62%)	0.072	16 (47%)	0.882
	Bilateral	45	35 (78%)		22 (49%)		41	33 (80%)		20 (49%)	
LD Meta	Absence	76	43 (57%)	0.016	28 (37%)	0.012	52	34 (65%)	0.055	21 (40%)	0.047
	Presence	28	23 (82%)		18 (64%)		23	20 (87%)		15 (65%)	
Platinum-resistance	Absence	62	38 (61%)	0.016	21 (34%)	0.014	48	32 (67%)	0.008	19 (40%)	0.043
	Presence	20	18 (90%)		13 (65%)		16	16 (100%)		11 (69%)	
CA19-9	Normal	66	47 (71%)	0.060	32 (48%)	0.158	50	39 (78%)	0.341	26 (52%)	0.156
	Elevated	17	8 (47%)		5 (29%)		8	5 (63%)		2 925%)	
CA125	Normal	18	7 (39%)	0.004	5 (28%)	0.121	7	4 (57%)	0.249	4 (57%)	0.630
	Elevated	75	56 (75%)		36 (48%)		61	47 (77%)		29 (48%)	
Histologic grade	Low (1)	27	5 (19%)	<0.001	6 (22%)	0.007	12	4 (33%)	0.001	4 (33%)	0.267
	High (2 & 3)	77	61 (79%)		40 (52%)		63	50 (79%)		32 (51%)	
Histologic type	Serous	75	54 (72%)	<0.001	36 (48%)	0.254					
	Mucinous	20	3 (15%)		5 (25%)						
	Endometrioid	5	5 (100%)		3 (60%)						
	Clear cell	3	3 (100%)		1 (33%)						
	Malignant Brenner	1	1 (100%)		1 (100%)						
BRCA1	Negative	58	28 (48%)	<0.001			39	22 (56%)	0.002		
	Positive	46	38 (83%)				36	32 (89%)			

LN; lymph node, LD Meta; latent distant metastasis.

The cases included in this study are heterogeneous. Various histologic types of ovarian carcinomas with different biologic and clinical background were included in this study. Thus, we did additional analysis according to the histologic types, especially for the serous carcinoma. Among the 75 serous carcinomas, DBC1 expression was significantly associated with higher histologic grade (*P* = 0.001) and platinum-resistance (*P =* 0.008) and showed a slight association of borderline significance with bilateral tumor (*P =* 0.072) and presence of latent distant metastasis (*P =* 0.055). BRCA1 expression was significantly associated with latent distant metastasis (*P =* 0.047) and platinum-resistance (*P =* 0.008) (Table 1).

### Expression of DBC1 and BRCA1 correlate with reduced overall survival and relapse-free survival in ovarian carcinomas by univariate analysis

**Figure 2:**
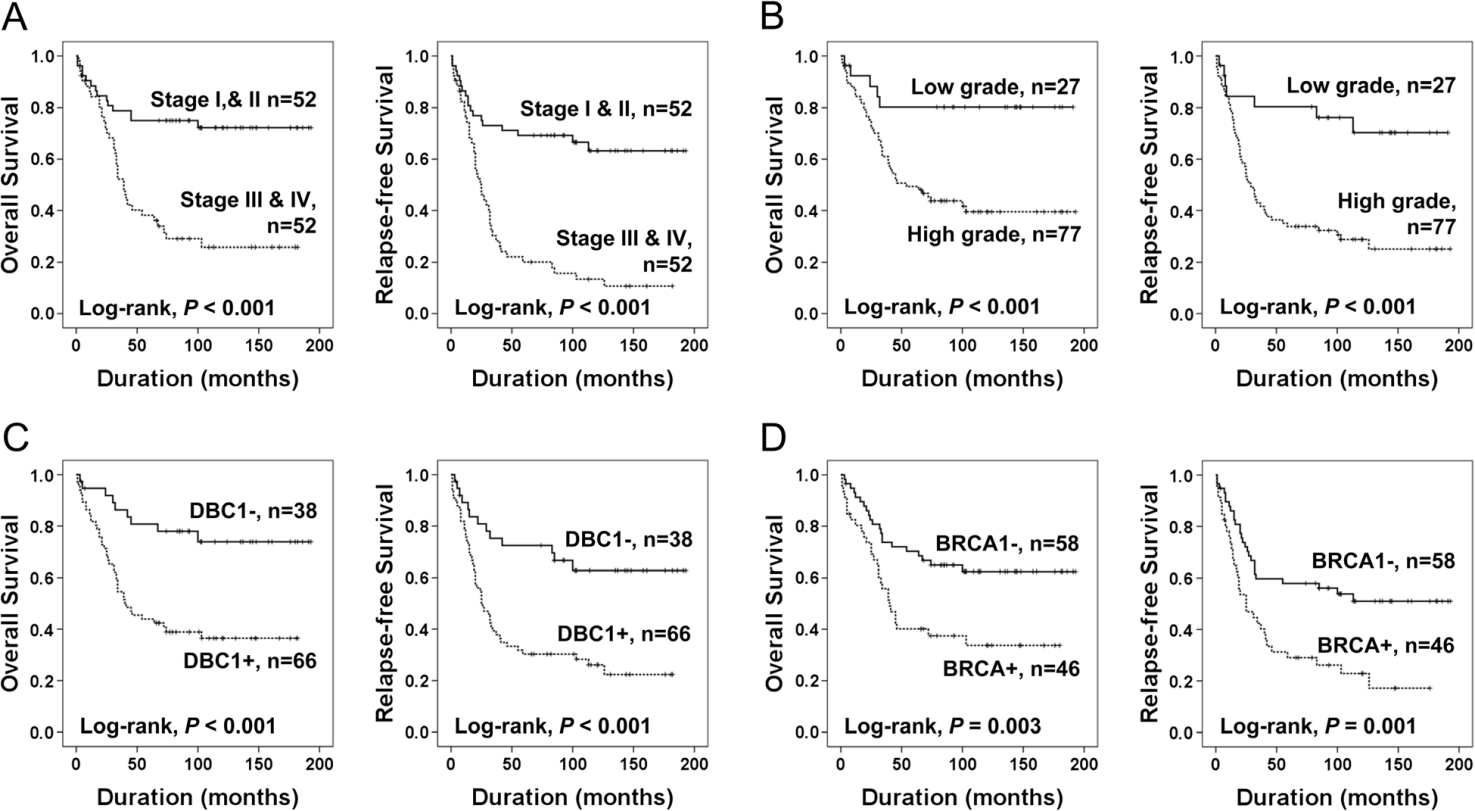
**Kaplan-Meier survival analyses in 104 overall ovarian carcinomas.** Overall survival and relapse-free survival according to the tumor stage **(A)**, histologic grade **(B)**, DBC1 expression **(C)**, and BRCA1 expression **(D)**.

Univariate analysis for the OS and RFS of the variable clinicopathological factors and DBC1 and BRCA1 expression in ovarian carcinomas are shown in Table 2. Among the 104 general cases of ovarian carcinoma, older age of the patients (OS; *P* < 0.001, RFS; *P* = 0.003), higher tumor stage (OS; *P* < 0.001, RFS; *P* < 0.001), presence of ascites (OS; *P* = 0.006, RFS; *P* = 0.017), increased pre-operative serum level of CA125 (OS; *P* = 0.013, RFS; *P* = 0.004), histologic grade (OS; *P* = 0.005, RFS; *P* < 0.001), DBC1 expression (Log-rank, OS; *P* < 0.001, RFS; *P* < 0.001), and BRCA1 expression (Log-rank, OS; *P* = 0.003, RFS; *P* = 0.003) were associated with shorter OS and RFS (Table 2) (Figure 2). The patients with tumors expressing DBC1 had a 3.474-fold (*P <* 0.001, 95% confidence interval [95% CI]; 1.684-7.166) greater risk of death and a 3.007-fold (*P <* 0.001, 95% CI; 1.624-5.567) greater risk of relapse or death. The five- and ten-year OS rates of the DBC1-negative group were 78% and 73%, respectively, and were only 42% and 36% in DBC1-positive group, respectively. In addition, among the 83 patients who received adjuvant chemotherapy, DBC1 expression was significantly associated with shorter OS (*P* = 0.004, hazard ration [HR]; 3.625, 95% CI; 1.518-8.656) and RFS (*P* = 0.004, HR; 2.738, 95% CI; 1.368-5.477). BRCA1 expression predicted shorter OS (*P* = 0.005, HR; 2.263, 95% CI; 1.287-3.979) and RFS (*P* = 0.002, HR; 2.254, 95% CI; 1.359-3.739) (Table 2).

**Table 2:** **Univariate Cox regression analyses for overall survival and relapse-free survival in ovarian carcinomas**

**Characteristics**		**No.**	**OS**		**RFS**	
			**HR (95% CI)**	***P***	**HR (95% CI)**	***P***
Overall ovarian carcinomas (n = 104)						
	Age, y, ≥ 60 (*vs* < 60)	33/104	2.686 (1.535-4.698)	<0.001	2.157 (1.299-3.582)	0.003
	Stage, III & IV (*vs* I & II)	52/104	3.579 (1.917-6.681)	<0.001	3.930 (2.245-6.878)	<0.001
	Tumor size, cm, > 10 (*vs* ≤ 10)	36/104	0.524 (0.274-1.004)	0.051	0.649 (0.375-1.125)	0.123
	LN metastasis, presence (*vs* absence)	20/104	1.568 (0.831-2.959)	0.165	1.889 (1.076-3.314)	0.027
	Ascites, presence (*vs* absence)	33/104	1.997 (1.140-3.498)	0.016	1.854 (1.116-3.077)	0.017
	Bilaterality, bilateral (*vs* unilateral)	45/104	1.647 (0.943-2.876)	0.080	1.995 (1.204-3.303)	0.007
	CA19-9, elevated (*vs* normal)	17/83	0.753 (0.314-1.806)	0.525	0.811 (0.379-1.733)	0.588
	CA125, elevated (*vs* normal)	75/93	4.451 (1.376-14.393)	0.013	4.458 (1.609-12.351)	0.004
	Histologic grade, high (*vs* low)	77/104	3.762 (1.491-9.496)	0.005	3.794 (1.719-8.374)	<0.001
	DBC1, positive (*vs* negative)	66/104	3.474 (1.684-7.166)	<0.001	3.007 (1.624-5.567)	<0.001
	BRCA1, positive (*vs* negative)	46/104	2.263 (1.287-3.979)	0.005	2.254 (1.359-3.739)	0.002
Serous carcinomas (n = 75)						
	Age, y, ≥ 60 (*vs* < 60)	30/75	2.601 (1.411-4.796)	0.002	1.926 (1.116-3.323)	0.019
	Stage, III & IV (*vs* I & II)	46/75	2.263 (1.131-4.528)	0.021	2.809 (1.485-5.312)	0.001
	Tumor size, cm, > 10 (*vs* ≤ 10)	19/75	0.625 (0.289-1.352)	0.233	0.817 (0.436-1.532)	0.529
	LN metastasis, presence (*vs* absence)	19/75	1.200 (0.613-2.349)	0.595	1.502 (0.831-2.714)	0.178
	Ascites, presence (*vs* absence)	30/75	1.793 (0.977-3.291)	0.060	1.645 (0.953-2.839)	0.074
	Bilaterality, bilateral (*vs* unilateral)	41/75	1.200 (0.647-2.224)	0.563	1.423 (0.813-2.492)	0.217
	CA19-9, elevated (*vs* normal)	8/58	0.679 (0.206-2.236)	0.524	1.086 (0.425-2.773)	0.863
	CA125, elevated (*vs* normal)	61/68	2.898 (0.697-12.056)	0.144	2.152 (0.668-6.931)	0.199
	Histologic grade, high (*vs* low)	63/75	4.401 (1.061-18.260)	0.041	4.341 (1.347-13.990)	0.014
	DBC1, positive (*vs* negative)	54/75	4.277 (1.674-10.926)	0.002	2.811 (1.363-5.794)	0.005
	BRCA1, positive (*vs* negative)	36/75	1.861 (1.007-3.438)	0.047	1.792 (1.032-3.110)	0.038
High-grade serous carcinomas (n = 63)						
	Age, y, ≥ 60 (*vs* < 60)	27/63	2.090 (1.119-3.900)	0.021	1.591 (0.907-2.791)	0.105
	Stage, III & IV (*vs* I & II)	38/63	2.188 (1.080-4.434)	0.030	2.811 (1.464-5.399)	0.002
	Tumor size, cm, > 10 (*vs* ≤ 10)	15/63	0.736 (0.339-1.599)	0.439	0.860 (0.448-1.651)	0.650
	LN metastasis, presence (*vs* absence)	15/63	1.520 (0.766-3.017)	0.232	1.841 (0.992-3.417)	0.053
	Ascites, presence (*vs* absence)	26/63	1.395 (0.749-2.598)	0.294	1.340 (0.764-2.351)	0.308
	Bilaterality, bilateral (*vs* unilateral)	36/63	1.300 (0.683-2.475)	0.424	1.452 (0.809--2.603)	0.211
	CA19-9, elevated (*vs* normal)	6/49	0.726 (0.220-2.403)	0.600	1.344 (0.521-3.467)	0.541
	CA125, elevated (*vs* normal)	53/58	2.220 (0.531-9.276)	0.275	1.443 (0.446-4.672)	0.540
	DBC1, positive (*vs* negative)	50/63	4.031 (1.427-11.382)	0.008	2.540 (1.135-5.684)	0.023
	BRCA1, positive (*vs* negative)	32/63	2.010 (1.063-3.803)	0.032	1.719 (0.973-3.038)	0.062

**Figure 3:**
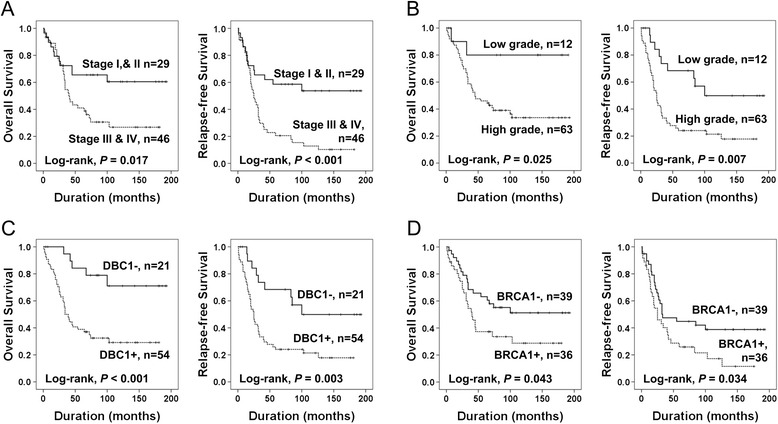
**Kaplan-Meier survival analyses in 75 ovarian serous carcinomas.** Overall survival and relapse-free survival according to the tumor stage **(A)**, histologic grade **(B)**, DBC1 expression **(C)**, and BRCA1 expression **(D)**.

When we did additional analysis in the subpopulation of serous carcinomas, the factors significantly associated with both OS and RFS by univariate analysis were the age of patients (OS; *P* = 0.002, RFS; *P* = 0.019), tumor stage (OS; *P* = 0.021, RFS; *P* = 0.001), histologic grade (OS; *P* = 0.041, RFS; *P* = 0.014), and DBC1 expression (Log-rank, OS; *P* < 0.001, RFS; *P* = 0.003), and BRCA1 expression (Log-rank, OS; *P* = 0.043, RFS; *P* = 0.034) (Table 2) (Figure 3). The patients who had DBC1-expressing serous carcinoma had a 4.277-fold (*P =* 0.002, 95% CI; 1.674-10.926) greater risk of death and its expression was significantly associated with shorter RFS (*P =* 0.005, HR; 2.811, 95% CI; 1.363-5.794) (Figure 3C).

**Figure 4:**
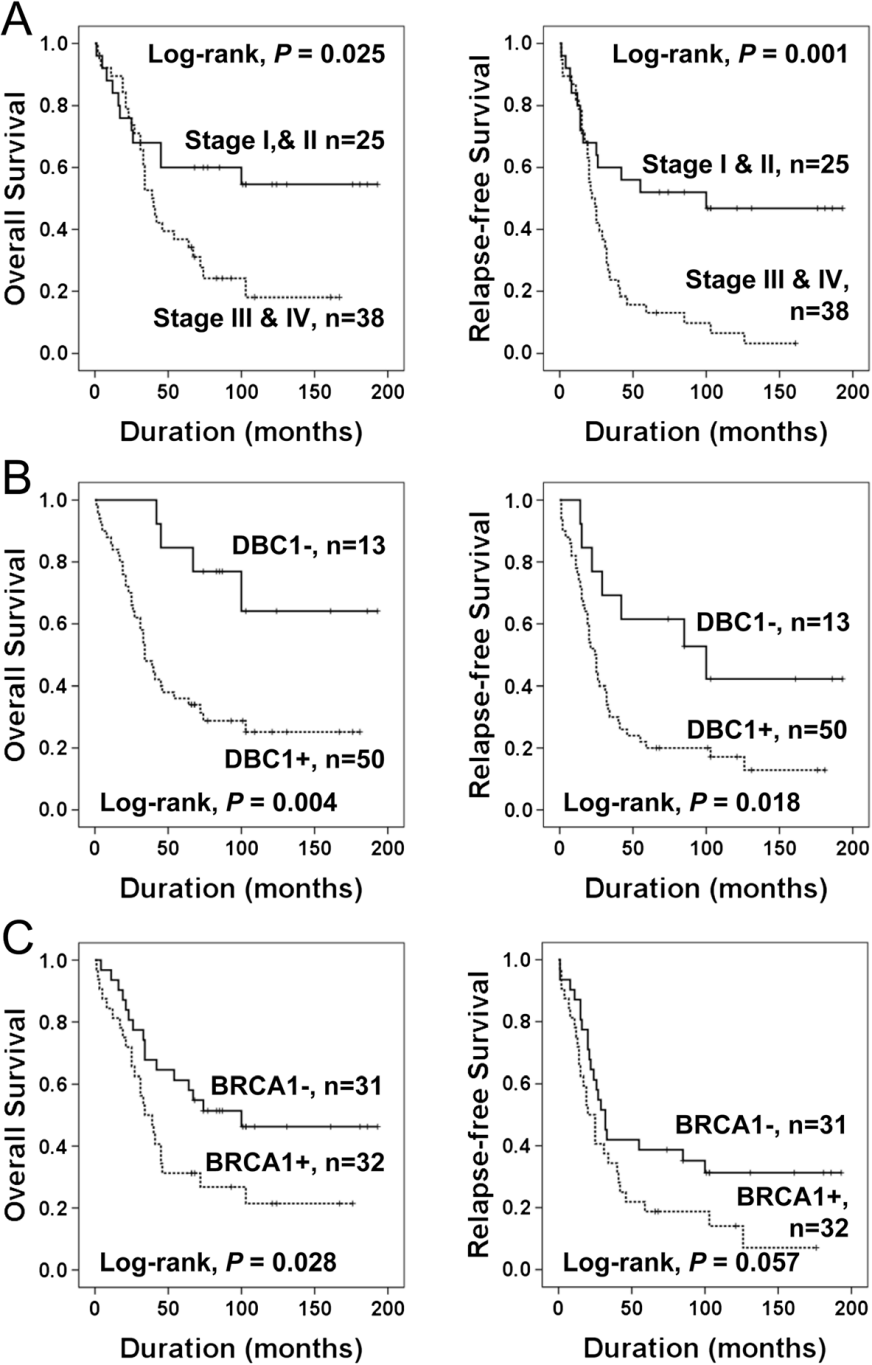
**Kaplan-Meier survival analyses in 63 high-grade serous carcinomas.** Overall survival and relapse-free survival according to the tumor stage **(A)**, DBC1 expression **(B)**, and BRCA1 expression **(C)**.

Among the 63 high-grade serous carcinomas, tumor stage and the expression of DBC1 were significantly associated with both OS and RFS, and the age of patients and BRCA1 expression were significantly associated with OS (Table 2) (Figure 4). The expression of DBC1 predicted a 4.031-fold (*P =* 0.008, 95% CI; 1.427-11.382) greater risk of death and a 2.540-fold (*P =* 0.005, 95% CI; 1.135-5.684) greater risk of relapse or death of high-grade serous carcinoma patients (Table 2). The OS rates at five- and ten-years of DBC1-negative high-grade serous carcinomas were 85% and 64%, respectively, and were 36% and 25% in DBC1-positive high-grade serous carcinomas.

In the 20 cases of mucinous carcinomas, tumor stage was significantly associated with shorter OS (Log-rank, *P <* 0.001) and RFS (Log-rank, *P <* 0.001). DBC1 expression showed borderline significance for the prediction of OS of mucinous carcinoma patients (Log-rank, *P =* 0.060).

### Expression of DBC1 is an independent prognostic indicator of worse survival outcome in ovarian carcinomas by multivariate analysis

The factors significantly associated with OS or RFS by univariate analysis were included in the multivariate analysis. Because of data for CA125 was missing in 11 patients, the CA125 level was not included in the multivariate analysis. The factors included in the multivariate analysis of OS and RFS were age, tumor stage, lymph node metastasis, presence of ascites, bilaterality of the tumor, histologic grade, BRCA1 expression, and DBC1 expression. In 104 ovarian carcinomas, the factors significantly associated with OS by multivariate analysis were age (*P* = 0.010), tumor stage (*P* = 0.006), and DBC1 expression (*P* = 0.021). The patients with tumors expressing DBC1 had a 2.423 fold (95% CI, 1.144-5.132) greater risk of death (Table 3). Tumor stage (*P <* 0.001) and histologic grade (*P =* 0.007) were the independent predictor of RFS. In addition, DBC1 expression was an independent predictor of OS (*P* = 0.026, HR; 2.735, 95% CI; 1.128-6.634) among the 83 patients who received adjuvant chemotherapy, as indicated by multivariate analysis.

**Table 3:** **Multivariate Cox regression analyses for overall survival and relapse-free survival in ovarian carcinomas**

**Characteristics**	**OS**		**RFS**	
	**HR (95% CI)**	***P***	**HR (95% CI)**	***P***
Overall ovarian carcinomas^a^				
Age, y, ≥ 60 (*vs* < 60)	2.100 (1.191-3.702)	0.010		
Stage, III & IV (*vs* I & II)	2.488 (1.295-4.780)	0.006	3.426 (1.944-6.036)	<0.001
Histologic grade, high (*vs* low)			3.023 (1.355-6.742)	0.007
DBC1, positive (*vs* negative)	2.423 (1.144-5.132)	0.021		
Serous carcinomas^b^				
Age, y, ≥ 60 (*vs* < 60)	2.241 (1.212-4.144)	0.010		
Stage, III & IV (*vs* I & II)			3.023 (1.588-5.755)	<0.001
Histologic grade, high (*vs* low)			4.818 (1.487-15.614)	0.009
DBC1, positive (*vs* negative)	3.757 (1.462-9.653)	0.006		
High-grade serous carcinomas^c^				
Age, y, ≥ 60 (*vs* < 60)	1.961 (1.050-3.664)	0.035		
Stage, III & IV (*vs* I & II)			2.488 (1.280-4.836)	0.007
DBC1, positive (*vs* negative)	3.828 (1.353-10.827)	0.011	2.059 (0.907-4.677)	0.084

^a^The variables included in the multivariate analysis were age, tumor stage, lymph node metastasis, presence of ascites, bilaterality of the tumor, histologic grade, BRCA1 expression, and DBC1 expression. ^b^The variables included in the multivariate analysis were age, tumor stage, histologic grade, BRCA1 expression, and DBC1 expression. ^c^The variables included in the multivariate analysis were age, tumor stage, BRCA1 expression, and DBC1 expression.

In the subpopulation of 75 serous carcinomas, the age of the patients (*P* = 0.010) and DBC1 expression (*P* = 0.006) were the independent predictors of OS. The expression of DBC1 predicted a 3.757-fold (95% CI, 1.462-9.653) greater risk of death. The factors significantly associated with RFS in serous carcinoma were tumor stage (*P <* 0.001) and histologic grade (*P =* 0.009) (Table 3). In addition, the age of the patients (*P* = 0.035) and the expression of DBC1 (*P* = 0.011) were the independent predictor of OS in high-grade serous carcinomas. The expression of DBC1 predicted a 3.828-fold (95% CI, 1.353-10.827) greater risk of death of high-grade serous carcinoma patients (Table 3).

## Discussion

This study has shown that immunohistochemical expression of DBC1 was significantly associated with advanced clinicopathological factors of ovarian carcinoma such as higher tumor stage, latent distant metastasis, platinum-resistance, elevated serum level of CA125, and higher histologic grade. Moreover, DBC1 expression was significantly associated with shorter survival of ovarian carcinomas, especially in high-grade serous carcinomas. In agreement with our results, increased expression of DBC1 has been reported as an indicator of poor prognosis of gastric carcinoma [15], breast carcinoma [14], colorectal carcinoma [9], esophageal carcinoma [20], clear cell renal cell carcinoma [12], diffuse large B cell lymphoma [13], and soft tissue sarcomas [13]. Nevertheless, there has been no report investigating DBC1 expression in ovarian carcinomas. Therefore, this is the first report that examined DBC1 expression in human ovarian tumors and suggests that DBC1 expression might be usable as a prognostic indicator for ovarian carcinoma patients.

DBC1 is interesting because of its putative role for the inhibition of SIRT1 and has been suggested as tumor suppressor [4,16,32]. However, there have been conflicting reports regarding the role of DBC1 and SIRT1 in human cancers. SIRT1-induced expression of various oncogenes and formed a positive-feedback loop with the c-Myc oncogene to stimulate tumorigenesis [33,34]. However, SIRT1 formed a negative-feedback loop with the c-Myc oncogene in another report [35]. Moreover, the effects of the expression of DBC1 and SIRT1 in human malignant tumors varied according to cell type [6,9,34,36,37]. The expression of both DBC1 and SIRT1 predicted shorter survival of gastric carcinoma [15], breast carcinoma [14], clear cell renal cell carcinoma [12], soft-tissue sarcoma [13], and diffuse large B cell lymphoma [13,16]. In colon cancer, DBC1 was overexpressed in colorectal cancer and predicted shorter survival of patients [9]. In contrast, another study reported that SIRT1 expression is associated with poor prognosis but DBC1 expression is associated with favorable prognosis of gastric cancer patients [36]. Although the expression of SIRT1 was higher in ovarian carcinomas compared with benign and borderline ovarian tumors, SIRT1 expression was associated favorable prognosis of ovarian carcinoma patients [38]. Therefore, poor prognosis of DBC1-expressing ovarian carcinoma might be related to its inhibitory role for SIRT1. However, the relationship between DBC1 and SIRT1 was been frequently dissociated as shown in breast cancer [6]. Moreover, the knock-down of DBC1 inhibited proliferation of liver cancer cells [37] and suppressed invasiveness of gastric cancer cells [12]. Therefore, it may be likely that DBC1 has its own role in tumorigenesis. DBC1 regulates BRCA1-mediated function by binding to the BRCT domain in addition to the suppression of SIRT1 expression [22]. In addition, DBC1 inhibits senescence of premalignant cells by disrupting the SUV39H1-SIRT1 complex. However, DBC1 showed a co-inhibitory effect for SUV39H1 and SIRT1 [8]. Therefore, there is a possibility that DBC1 may have both tumorigenic and anti-tumorigenic roles [8,22]. Thus, further study is needed to explore the exact role of DBC1 in tumorigenesis.

Because the expression of DBC1 was positively correlated with higher tumor stage, higher tumor grade, and latent metastasis of ovarian carcinoma, there is a possibility that DBC1 might be involved in the acquisition of invasive and metastatic potential. Recently, it has been shown that DBC1 is associated with the invasive potential of esophageal squamous cell carcinoma [20] and is important in the EMT of gastric carcinoma cells [12]. Especially, the oncogenic role of DBC1 was regulated by the kinase effect of CK2α. CK2α phosphorylates DBC1 and that is important for the induction of EMT. Knockdown of DBC1 inhibited invasiveness of gastric cancer cells and a point mutation at the phosphorylation site of DBC1 decreased the expression of MMP2, MMP9, snail, smad3, and N-cadherin [12]. In addition, DBC1 induced anoikis resistance that is important in tumor metastasis by activating the NFkB signaling pathway in breast cancer [17].

The higher rate of distant metastatic relapse in DBC1-expressing ovarian carcinomas raises the possibility that DBC1 might be involved in the acquisition of resistance for the postoperative chemotherapies. Among the 83 ovarian carcinoma patients who received adjuvant chemotherapy, the expression of DBC1 predicted shorter OS and RFS. In addition, among the 55 high-grade serous carcinoma patients who received adjuvant chemotherapy, DBC1 expression was significantly associated with poor OS (*P* = 014, HR; 4.484, 95% CI; 1.362-14.758) and RFS (*P* = 0.042, HR; 2.471, 95% CI; 1.035-5.899). Moreover, DBC1 expression correlated with platinum-resistance. All serous carcinoma patients having tumors with DBC1 expression (100%, 16/16) showed platinum-resistance. In contrast, 62% (32/48) of DBC1-negative serous carcinoma patients showed platinum-resistance. Similarly, DBC1 expression was associated with frequent relapse and shorter survival of breast carcinoma patients who received adjuvant chemotherapy [14]. Although it did not reach statistical significance, lower expression of DBC1 would indicate a favorable pathological response to chemotherapy [39].

Because DBC1 is involved in the inhibition of BRCA1 [22] and *BRCA1/2* status is important in the development and progression of ovarian carcinomas, we investigated the immunohistochemical expression of BRCA1 in ovarian carcinomas. Although we could evaluate the immunohistochemical expression of BRCA1, a recent report has shown that there is a strong correlation between the immunohistochemical expression and molecular events in *BRCA1* [30]. In this study, BRCA1 expression was significantly associated with latent distant metastasis, platinum-resistance, and higher histologic grade. In addition, in agreement with previous reports [24,40,41], we have demonstrated that low-expression of BRCA1 is associated with poor survival of ovarian carcinomas. The reason why the patients with defective BRCA1/2 have a longer survival times compared with *BRCA1/2* wild-type carcinomas is related with *BRCA1/2*-defects in cells making them more sensitive to conventional chemotherapy, especially to the platinum-based chemotherapy [23,24,42]. Our results have also shown that BRCA1-positivity is significantly associated with increased platinum-resistance (Table 1). Thereby, several therapeutic applications according to the *BRCA1/2* status are under evaluation. Recently, PARP inhibitors have been reported as being specifically applicable to the treatment of *BRCA1/2*-defective cancers [43,44]. In our study, the expression of DBC1 and BRCA1 showed positive correlation and the expressions of both molecules was related with platinum-resistance and shorter survival of ovarian carcinoma patients. Based on the inhibitory role of DBC1 for the BRCA1 [22], the co-expressing pattern of these two molecules in the poor prognostic group of ovarian carcinomas is questionable and paradoxical as *BRCA1/2* defective cancers are more susceptible to therapy [23,24,42]. This phenomenon might be related with the diverse roles of DBC1 in tumorigenesis and further study is needed.

Another possible oncogenic role of DBC1 might be related with its role in the co-activation of nuclear receptors [18,19,21]. DBC1 co-activates estrogen receptor and androgen receptor (AR), which can be ligand-dependent or ligand-independent [18,19,21]. Although there was no significant correlation between the expression of DBC1 and estrogen receptor in breast carcinoma [14], significant positive correlations between the expression of DBC1 and AR have been reported in clear cell renal cell carcinoma [12] and diffuse large B cell lymphoma [11]. Especially, the expression of both DBC1 and AR predicted shorter survival of cancer patients [11,12,14-16]. Additionally, ovarian epithelium expressing AR and androgen induced proliferation of ovarian epithelial cells and inhibited cell death [45]. In ovarian high-grade serous carcinomas, immunohistochemical expression of AR correlated with the S-phase fraction and AR expression decreased with platinum-based chemotherapy [46]. These reports suggest that AR is involved in the ovarian carcinogenesis. Therefore, there is a possibility that DBC1 is involved in ovarian tumorigenesis with the interaction with AR and further study is needed.

Ovarian carcinomas are a heterogeneous group of cancers that have origins and pathogenic profiles that differ according to histologic types. Therefore, when we consider the prognostic impact of DBC1 expression according to histologic types of ovarian carcinoma, DBC1 predicted shorter survival in serous carcinomas, especially in high-grade serous carcinomas. The 10-years OS rate was only 25% in DBC1-positive cases, and was 64% in DBC1-negative cases. In mucinous carcinomas, DBC1 expression was very low compared with other subtypes of ovarian carcinomas (15% in mucinous carcinoma, 72% in serous carcinoma, and 100% in endometrioid carcinoma). However, despite the low frequency of DBC1-positivity and the small number of cases of mucinous carcinoma, DBC1 expression showed borderline significance in OS analysis (Log-rank, *P* = 0.060). The 10-years OS rates of DBC1-negative and DBC1-positive mucinous carcinomas were 76% and 33%, respectively. In line with our results, DBC1 expression was associated with a higher nuclear grade of breast carcinoma [39]. Therefore, although the expression rate of DBC1 differs according to the histologic types, our result suggests that DBC1 expression might be involved in the progression of ovarian carcinomas, regardless of histologic types. However, further study with a larger group of ovarian carcinoma is needed to clarify the role of DBC1 in ovarian carcinomas.

## Conclusions

In conclusion, to our knowledge, this is the first study to show that DBC1 is commonly expressed in ovarian carcinomas and its expression is predictive of prognosis of ovarian carcinoma patients, especially in high-grade serous carcinomas and possibly in mucinous carcinomas. In addition, DBC1 expression was significantly associated with BRCA1 expression and their expressions were related with resistance to platinum-based chemotherapy and poor prognosis of ovarian carcinoma patients. Therefore, these results indicate that the expression of DBC1 and BRCA1 might be used for the prediction of prognosis of ovarian carcinomas. Especially, DBC1 expression might be helpful for the prediction of the prognosis of high-grade serous carcinomas. In addition, these findings suggest that DBC1 and BRCA1could be potential therapeutic targets for the treatment of ovarian carcinomas according to the expression status of DBC1 and BRCA1. However, because of the limited number of non-serous cases of ovarian carcinoma subtypes in this study, further study with more cases is needed to clarify the roles of DBC1 and BRCA1 in various histologic subtypes of ovarian carcinomas.

## Abbreviations

AR: Androgen receptor
CCAR2: Cell cycle and apoptosis regulator 2
CI: Confidence interval
DBC1: Deleted in breast cancer 1
EMT: Epithelial-mesenchymal transition
HR: Hazard ration
LN: Lymph node
OS: Overall survival
RFS: Relapse-free survival
TMA: Tissue microarray
